# State and Force Estimation on a Rotating Helicopter Blade through a Kalman-Based Approach

**DOI:** 10.3390/s20154196

**Published:** 2020-07-28

**Authors:** Roberta Cumbo, Tommaso Tamarozzi, Pavel Jiranek, Wim Desmet, Pierangelo Masarati

**Affiliations:** 1 Siemens Digital Industries Software, Interleuvenlaan 68, 3001 Leuven, Belgium; roberta.cumbo@siemens.com (R.C.); tommaso.tamarozzi@siemens.com (T.T.); pavel.jiranek@siemens.com (P.J.); 2 KU Leuven, Department of Mechanical Engineering, Celestijnenlaan 300 B, 3001 Heverlee, Belgium; wim.desmet@kuleuven.be; 3 DMMS-D Core Lab, Flanders Make, 3001 Leuven, Belgium; 4 Dipartimento di Scienze e Tecnologie Aerospaziali, Politecnico di Milano, Via la Masa 34, 20156 Milano, Italy

**Keywords:** state and load estimation, inverse identification, Kalman filter, multibody modeling

## Abstract

The interaction between the rotating blades and the external fluid in non-axial flow conditions is the main source of vibratory loads on the main rotor of helicopters. The knowledge or prediction of the produced aerodynamic loads and of the dynamic behavior of the components could represent an advantage in preventing failures of the entire rotorcraft. Some techniques have been explored in the literature, but in this field of application, high accuracy can be reached if a large amount of sensor data and/or a high-fidelity numerical model is available. This paper applies the Kalman filtering technique to rotor load estimation. The nature of the filter allows the usage of a minimum set of sensors. The compensation of a low-fidelity model is also possible by accounting for sensors and model uncertainties. The efficiency of the filter for state and load estimation on a rotating blade is tested in this contribution, considering two different sources of uncertainties on a coupled multibody-aerodynamic model. Numerical results show an accurate state reconstruction with respect to the selected sensor layout. The aerodynamic loads are accurately evaluated in post-processing.

## 1. Introduction

The main rotor is the most significant component of any helicopter because it generates the needed lift supporting the weight of the entire rotorcraft and provides the control moments required for the execution of different maneuvers. The flexible rotating blades are constantly interacting with the aerodynamic flow and are thus the main source of vibratory loads. These excitations are transmitted to the rotor hub; consequently, their knowledge becomes of significant importance to preventing failures and other undesirable mechanical effects on the entire rotorcraft. Besides, the variability of the aerodynamic loads produced by the structure and flow interaction is of high complexity and cannot be easily predicted or generalized for the same rotorcraft in different flight conditions. Therefore, the problem of structural monitoring in helicopters covers many aspects of rotorcraft design and operations. Several methodologies have been proposed in the literature. This problem can be approached in two related fields of research: structural component monitoring and prediction of structural behavior or aerodynamic loads.

A review of existing helicopter rotor health monitoring systems and techniques is presented in [1]. The health and usage monitoring system (HUMS) and the rotor track and balance (RTB) system have been considered valuable instruments to increase failure detection information and flight safety. Nevertheless, they do not provide detailed information about the structural status of the rotor components. Ganguli et al. [2] studied the damage detection problem at the blade level, in terms of localized changes of structural, inertia and aerodynamic properties. In a subsequent study, Ganguli et al. [3] extended the previous work by presenting one of the first contributions on the usage of artificial neural network (ANN) algorithms applied to damage modeling. Despite the disadvantage of a large amount of data needed for training, ANNs appears in several works [4,5,6] in the field of loads and damage monitoring in the helicopter area. In the last few decades, these techniques have become widely used for force reconstruction [7,8] and aerodynamic flow interaction [9,10] problems. One of the main advantages of ANN is that no physical model is required behind the application of the methodology. When model-based techniques are employed, the accurate analysis of rotor blade loads needs a coupled computational fluid dynamics (CFD) and computational structural dynamics (CSD) simulation, as accurately reported in [11].

In recent years, focus is being placed on the combination of test and model solutions [12,13,14,15]. Among these techniques, the so-called blade shape sensing [14] has been demonstrated to be an efficient modal-based approach to reconstruct the shape of the blade, combining a limited set of measurements with assumed modal shapes. The optimal number and positions of sensors still constitute an open field of research, together with the application of the technique to more complex systems, such as a full helicopter rotor. The assembled structure is difficult or time-consuming to obtain in terms of structural modeling and aerodynamic load computation with respect to the level of accuracy. The intention of this contribution is to propose an approach able to perform appreciable prediction of structural/aerodynamic information saving time in the modeling phase. The proposed approach makes use of the estimators, and in particular, of the Kalman filter [16,17], which aims at solving a state space model taking into account the model and observation (e.g., measurements in a real test case) uncertainties. Besides the standard formulation, a robust Kalman filter has been also presented in the literature [18,19,20] to address the problem of state estimation by being robust against the model’s uncertainty. Indeed, when some unknown modeling errors exist, the estimation of the standard Kalman filter might be not enough accurate. The robust Kalman filter is instead designed to consider all possible uncertainties [18] by modeling an energy bounded noise, against the stochastic white noise in the standard formulation [19]. The solution of two algebraic Riccati equations [18] is needed in the robust Kalman filter and this might be a disadvantage. The modeling of the uncertain system is also constrained to a norm-bound value, which has conceptually the same role of the model covariance matrix as the standard Kalman filter [16]. Some works (e.g., [21,22]) already report the usage of the Kalman filter for damage detection in rotorcraft applications. Different formulations of the standard Kalman filter (KF) are widely used in the literature, such as the extended Kalman filter (EKF) and the unscented Kalman filter (UKF) [16]. The EKF is one of the possible non-linear versions of the Kalman filter. The main principle is the linearization of the non-linear system around the current estimated state using a first-order truncation of the Taylor series expansion of the model equations. Some references on the application of the EKF can be found in [23,24,25]. The UKF is instead a recursive estimator that addresses some of the approximation issues of the EKF. The UKF does not explicitly approximate the nonlinear process and observation models; it uses instead the true nonlinear models and approximates the distribution of the state random variable. However, in many application cases, the EKF becomes more efficient from a computational point of view. An accurate comparison between EKF and UKF can be found in [24]. When a joint states/inputs estimation is performed, both EKF and UKF can be employed in a variant way. In this context, two different approaches are presented in the literature: the dual Kalman filter (DKF) [26] and the augmented Kalman filter (AKF) [16]. The DKF operates in two estimation stages, while the AKF estimates the states and the inputs simultaneously by introducing an augmented state vector and this defines a reduction of the computational cost. The AKF has been applied in many fields and was recently demonstrated to give accurate results for automotive applications [25,27]. The results showed that, when the adopted structural model was not accurate enough, the AKF still provided a good estimation of the states at the expense of the loads’ accuracy. Given the dependence of the aerodynamic loads on the states of the rotor blades [28], the proposed approach performs a multiple-load and state estimation, with the aim of reaching high accuracy for the states of the blades and re-using this information to compute the aerodynamic loads in post-processing.

As already stated, the Kalman filtering technology has been widely applied in a lot of research, but the intent of the authors is to demonstrate its potential in the rotorcraft field for load prediction, thereby exploring the advantages in the model complexity and needed sensor data. A multibody model [29] of the blades has been developed, following the finite segment formulation [30] approach. The model can be considered as simplified because the blade flexibility is not modeled with high accuracy like in a finite element modeling approach. This is, anyway, an acceptable simplification for quasi-steady flight conditions and also if the user is interested mainly in aerodynamic loads instead of blade deformations. Increasing the complexity of the model is out of the scope of this work, but it is the intent of the authors to investigate the proposed methodology on highly accurate rotor models in the future. Given the non-linear nature of a general multibody model, the EKF formulation has been adopted in its augmented formulation. A steady flight condition is investigated in this contribution. The 2-blade rotor model is first validated by comparing its results with those obtained using MBDyn—free, general-purpose multibody software [31]—and then used to predict aerodynamic load distribution and the displacement/velocity field along the blade span. This paper is structured as follows. In Section 2, the theoretical background is provided, giving a generic overview on all the touched-upon topics with the formulations used: rotor multibody modeling, aerodynamic modeling and augmented Kalman filter. In Section 3, the developed workflow needed for states and loads’ prediction is presented, followed by a numerical validation of the developed model and numerical results of the estimation problem in Section 4. Concluding remarks are discussed in Section 5.

## 2. Methodologies: Theoretical Background

In the present contribution, the structural model of the rotating blades is developed using a multibody simulation approach [29] based on the finite segment theory presented in [30]. A quasi-steady formulation is adopted for the aerodynamic loads and a uniform inflow model is used, based on the blade element momentum theory (BEMT) [32]. The prediction of aerodynamic loads and states of the developed multibody model is proposed using a Kalman-based approach.

### 2.1. Multibody Structural Model

In structural modeling, a multibody dynamics simulation is a convenient approach when the system is complex and phenomena caused by nonlinearities cannot be neglected. A rotating blade is subject to high stresses and deformations and is connected to the hub through some mechanisms, e.g., pitch, flap and lag hinges, which need to be modeled in order to reproduce the correct behavior of the fully assembled structure. There are two main classes of formulations for multibody analysis: ordinary differential equations (ODE) and differential-algebraic equations (DAE). A detailed explanation of the differences between the two formulations is provided in [33,34]. In this contribution, an index-2 DAE approach is chosen, which formulates the equations of motion of a constrained system as:

(1) $$\begin{array}{c} \left\{\begin{array}{c} \dot{q}\left(t\right)-v\left(t\right)=0\\ M\left(q\left(t\right)\right)\dot{v}\left(t\right)+f\left(q\left(t\right),v\left(t\right),t\right)-B{\left(q\left(t\right)\right)}^{T}\lambda_{s}\left(t\right)=0\\ B(q(t))v(t)=0 \end{array}\right. \end{array}$$

where *t* is the time variable; $q\in R^{n}$ and $v\in R^{n}$ are the *n*-coordinates and *n*-velocity vectors of the system; $M\in R^{n\times n}$ is the mass matrix; $f\in R^{n}$ is a general non-linear term including all the other forces, e.g., elastic, external and quadratic contributions; $B\in R^{n\times m}$ is the Jacobian matrix of the constraints, given by differentiating the constraint condition with respect to q; $\lambda_{s}\in R^{m}$ is the vector of *m* Lagrange multipliers; and $\dot{q}$ and $\dot{v}$ are the time derivatives of q and v. The set of Equations in (1) includes only the velocity constraint. A way to enforce the index-2 formulation is to use a variant form given by Gear, Gupta and Leimkuhler (GGL) [35]:

(2) $$\begin{array}{c} \left\{\begin{array}{c} \dot{q}\left(t\right)-v\left(t\right)+B{\left(q\left(t\right)\right)}^{T}\mu_{s}\left(t\right)=0\\ M\left(q\left(t\right)\right)\dot{v(t)}+f\left(q\left(t\right),v\left(t\right)\right)-B{\left(q\left(t\right)\right)}^{T}\lambda_{s}\left(t\right)=0\\ B(q(t))v(t)=0\\ g(q(t))=0 \end{array}\right. \end{array}$$

In Equation (2), additional multipliers $\mu_{s}$ at velocity level and a set of algebraic equations $g\left(q\left(t\right)\right)\in R^{m}$, i.e., position constraints, are included. The GGL formulation is the one adopted in the multibody solver used in this work. For sake of simplicity, the time dependence of all variables will be omitted in the next sections.

#### 2.1.1. Finite Segment Beam Formulation

**Figure 1:**
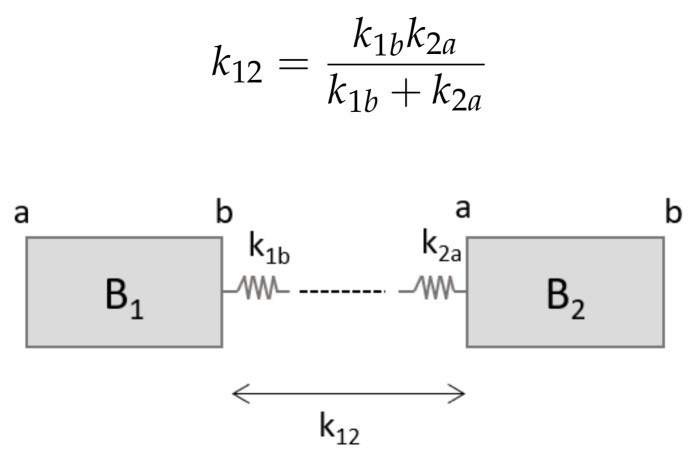
Two rigid bodies connected through elastic elements. *a* and *b* are the two faces of each body where the connections are. $k_{12}$ is the total stiffness matrix given by the interaction of both elastic forces from faces $B_{1b}$ and $B_{2a}$.

Each multibody model of the blades is composed by $N_{e}$ rigid bodies, connected by $N_{e}-1$ flexible elements. In the finite segment approach [30], these are spring elements, as shown in Figure 1. Given two bodies $B_{1}$ and $B_{2}$, the equivalent stiffness $k_{12}$ is:

(3) $$\begin{array}{c} k_{12}=\frac{k_{1b}k_{2a}}{k_{1b}+k_{2a}} \end{array}$$

A rigid body in space has six degrees of freedom (DOFs), i.e., three positions and three rotations; thus, the stiffness parameter in Equation (3) results in a 6 by 6 matrix $k_{12}$. For a certain body *j*, the elemental stiffness matrix at the generic face *f* can be derived from a finite element beam theory [36] formulation:

(4) $$\begin{array}{cc} k_{jf} & =\left[\begin{array}{cccccc} \frac{EA}{l} & 0 & 0 & 0 & 0 & 0\\ 0 & \frac{12EI_{z}}{l^{3}\left(1+\Phi_{y}\right)} & 0 & 0 & 0 & \frac{-6EI_{y}}{l^{2}\left(1+\Phi_{z}\right)}\\ 0 & 0 & \frac{12EI_{y}}{l^{3}\left(1+\Phi_{z}\right)} & 0 & \frac{6EI_{z}}{l^{2}\left(1+\Phi_{y}\right)} & 0\\ 0 & 0 & 0 & \frac{GJ}{l} & 0 & 0\\ 0 & 0 & \frac{6EI_{z}}{l^{2}\left(1+\Phi_{y}\right)} & 0 & \frac{\left(4+\Phi_{z}\right)EI_{y}}{l(1+\Phi_{z})} & 0\\ 0 & \frac{-6EI_{z}}{l^{2}\left(1+\Phi_{z}\right)} & 0 & 0 & 0 & \frac{\left(4+\Phi_{y}\right)EI_{z}}{l(1+\Phi_{y})} \end{array}\right] \end{array}$$

where *l* is the free-length of the beam; EA, $EI_{y}$, $EI_{z}$ and GJ are the cross-sectional properties of the beam; and $\Phi_{y}$ and $\Phi_{z}$ are two coefficients to account for shear deformation effects.

### 2.2. Aerodynamic Model

The interaction between a rotating blade and the external fluid defines a complex aerodynamic field and a 3D analysis should be used to enable an accurate performance estimation of the rotor. However, this contribution aims to outline a general workflow for loads and states prediction on a rotating blade in a realistic aerodynamic scenario. The employment of highly accurate aerodynamic modeling, such as CFD analysis, is thus out of the scope of this work. In this paper, MBDyn is first used as the reference code for validation purpose and then to generate reference data for Kalman filtering estimation. A 2D aerodynamic model is employed, computing the inflow velocity based on the BEM theory. An advantage that will be underlined in Section 3 is the dependence of the aerodynamic loads on the states of the blade structural model. All the analyses presented in the paper refer to a quasi-steady aerodynamic model.

#### 2.2.1. Aerodynamic Loads

**Figure 2:**
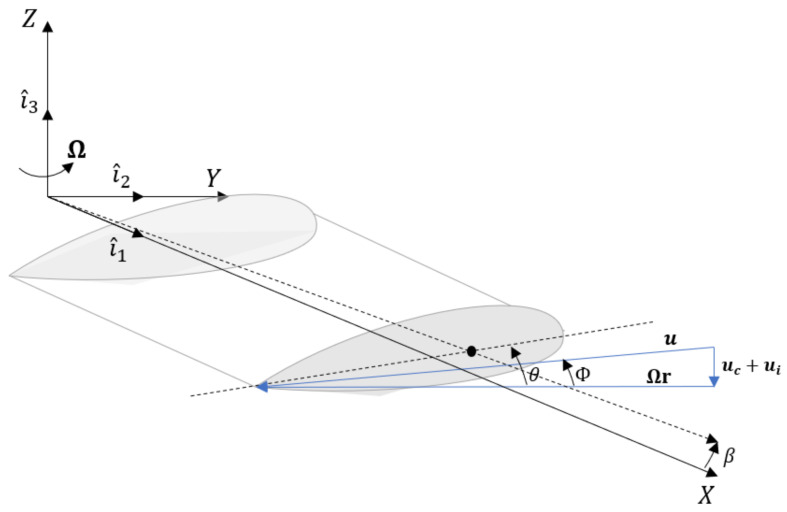
Velocity vector and angles definition for a blade station at distance *r* from the hub rotating frame.

Figure 2 shows a sketch of an airfoil in the rotating reference frame which is adopted in the presented rotor model. It is identified by {X,Y,Z} with corresponding unit vectors $\left\{{\hat{i}}_{1},{\hat{i}}_{2},{\hat{i}}_{3}\right\}$. In the helicopter modeling, the rotating frame follows the motion of the hub, which rotates with rotational speed Ω. The elemental lift dL and drag dD, evaluated at the stage r, can be written as:

(5) $$\begin{array}{c} dL=\frac{1}{2}\rho {\dot{q}}_{r}^{T}{\dot{q}}_{r}C_{L}cdr\\ dD=\frac{1}{2}\rho {\dot{q}}_{r}^{T}{\dot{q}}_{r}C_{D}cdr \end{array}$$

dL and dD are respectively orthogonal and parallel to the air velocity u. From Equation (5):

- ρ is the air density.
- ${\dot{q}}_{r}$ is the velocity vector evaluated at the stage r. Considering the blade modeled as a rigid body, ${\dot{q}}_{r}$ is expressed as:
  (6) $$\begin{array}{c} {\dot{q}}_{r}=\Omega {\hat{i}}_{3}\times r+\dot{\beta }\left|r\right|{\hat{i}}_{3}+\left(u_{1}{\hat{i}}_{1}+u_{2}{\hat{i}}_{2}+u_{3}{\hat{i}}_{3}\right) \end{array}$$
  where $\dot{\beta }$ is the flapping velocity, which is defined as the time-derivative of the angle β shown in Figure 2. $u_{1,2,3}$ are the components of the air velocity. In hovering flight, the air velocity is reduced to only the vertical contribution along ${\hat{i}}_{3}$, naming the inflow velocity $u_{i}$. If the point resides on an axis parallel to ${\hat{i}}_{1}$, then:
  (7) $$\begin{array}{c} {\dot{q}}_{r}=\Omega |r|{\hat{i}}_{2}+\dot{\beta }\left|r\right|{\hat{i}}_{3}+\left(u_{1}{\hat{i}}_{1}+u_{2}{\hat{i}}_{2}+u_{3}{\hat{i}}_{3}\right). \end{array}$$
- $C_{L}$ and $C_{D}$ are the aerodynamic coefficients; in general, they are tabular values for a given airfoil. Important for the evaluation of the total thrust produced by the rotating blades is the expression of the lift coefficient $C_{L}$. This contribution focuses on small angles of attack α and low values of the Mach number, such that a linear relation $C_{L}=a\alpha$ is guaranteed, with *a* representing the lift slope of the section, and α=θ−Φ (Figure 2). θ and Φ are respectively the pitch and inflow angles of the section. More details about the derivation of Φ are given in Section 2.2.2.
- *c* indicates the chord of the blade at stage *r*.

In this work, the assumption of constant slope *a* and angle Φ for all the points of a given section is made. The elemental lift in Equation (5) can be rewritten as:

(8) $$\begin{array}{c} dL=\frac{1}{2}\rho {\dot{q}}_{r}^{T}{\dot{q}}_{r}a\left(\theta -\Phi \right)cdr \end{array}$$

By integrating the elemental lift along the radius *R*, the total thrust produced by a rotor with *b* blades at time *t* is:

(9) $$\begin{array}{c} T=\frac{1}{2}\rho ab\Omega^{2}\int_{0}^{R}c\left(\theta -\Phi \right)r^{2}dr \end{array}$$

Further details can be found in reference [37].

#### 2.2.2. Uniform Inflow Model

In the previous section, the inflow velocity $u_{i}$ was introduced. It indicates the contribution of the volume of air that is moved by the rotor on the blade velocity. An inflow model based on the BEM theory is used with the assumption of uniform distribution, which is an acceptable approximation for hovering or in general in quasi-steady flight conditions. The inflow velocity can thus be written as:

(10) $$\begin{array}{c} u_{i}=\sqrt{\frac{T}{2\rho A}} \end{array}$$

with $A=\pi R^{2}$ being rotor disk area. In the following, we will refer to the induced velocity as its dimensionless value $\lambda_{i}=\frac{u_{i}}{\Omega R}$. The sectional inflow angle becomes:

(11) $$\begin{array}{c} \Phi =\frac{u_{i}}{\Omega r}=\frac{\lambda_{i}}{x} \end{array}$$

with x=r/R, i.e., the dimensionless spanwise position of the blade section. From Equation (10), it is clear that the value of the induced velocity is constantly updated depending on the value of the thrust produced at time *t*. Such dependence drops for a steady condition, but it is still important, for this work, to take into account time-dependent conditions, e.g., the transient regime from initial conditions to steady-state. Dynamic inflow models (e.g., Pitt–Peters, Peters–He [38]) are available, but they are not necessary in our estimation, as they mainly address very low frequency transients usually associated with flight dynamics, rather than vibrations. Since the thrust *T* depends on the inflow angle (Equation (9)), an implicit problem needs to be solved iteratively. Two numerical approaches are presented in the literature [39], based on fixed-point or Newton–Raphson iteration. The latter is used in this contribution for the aerodynamic solver. It is reported in the following because of its importance in understanding the full aerodynamic model and the Kalman estimation principle. Introducing μ as the dimensionless air velocity parallel to the rotor disk, i.e., $\mu =\frac{|u|cos\alpha }{\Omega r}$ in the plane of rotation, the following implicit function is defined:

(12) $$\begin{array}{c} f\left(\lambda \right)=\lambda -\frac{C_{T}\left(\lambda \right)}{2\sqrt{\mu^{2}+\lambda^{2}}}=0 \end{array}$$

with $\lambda =\mu \tan\left(\alpha \right)-\lambda_{i}$. $C_{T}$ is the blade-loading coefficient or dimensionless thrust, defined as: $C_{T}=\frac{T}{\rho A{\left(\Omega R\right)}^{2}}$. Equation (12) is solved with the Newton–Raphson iteration scheme, and for small values of μ, the usage of the hover value as a first guess of λ, i.e., $\lambda_{0}=\sqrt{\frac{C_{T}}{2}}$, generally allows it to converge with a limited number of iterations.

### 2.3. Kalman Filter for Implicit Scheme

This section aims to give an overview of the main principle of the Kalman filter (KF) and to understand how and in which formulation it can be applied to a DAE system—for instance, Equation (2). The filter operates on a state-space form in two phases, i.e., prediction and correction. The model predicts the state in the next time instant and the same prediction is then corrected using the observations given as input to the algorithm at each time step. These two steps for a linear time invariant (LTI) system are translated in the state-space system:

(13) $$\begin{array}{c} \left\{\begin{array}{c} \dot{x}=Ax+Bp\\ y=Cx+Dp \end{array}\right. \end{array}$$

where x, p and y are respectively the state, input and observation vectors. A and B are matrices depending on the dynamics of the system; C and D depend on the nature of the observations. If the problem presents non-linearities, the system (13) becomes a set of non-linear implicit functions:

(14) $$\begin{array}{c} \left\{\begin{array}{c} h(x,\dot{x},p)=0\\ y=k(x,p) \end{array}\right. \end{array}$$

This set of equations can be easily identified in the GGL form in Equation (2) of a multibody system, defining the following state vector and its time derivative:

(15) $$\begin{array}{c} x={\left[q,v,\lambda_{s},\mu_{s}\right]}^{T} \end{array}$$

(16) $$\begin{array}{c} \dot{x}={\left[\dot{q},\dot{v},{\dot{\lambda }}_{s},{\dot{\mu }}_{s}\right]}^{T} \end{array}$$

A mismatch between Equations (14) and (2) can be observed because no information about the input vector p is included in the GGL set of equations. The dynamics of p should be added. Given the unknown nature of this entity in the estimation context, a good assumption is that of zero^th^-order dynamics, i.e., zero time-derivative of p, which leads to $\dot{p}=0$. This model has been widely adopted and validated in the literature [25,27,40,41], and a clearer explanation will be provided in the following. In this contribution, the estimation process is performed in discrete-time domain, and thus the discretization of the continuous-time GGL formulation expressed in Equation (2) is needed. The multibody solver used in the present work executes this step by applying the implicit backward differentiation formulas (BDF) integration method [42]:

(17) $$\begin{array}{c} {\dot{z}}_{k+1}=\sum_{j=0}^{s}\alpha_{j}z_{k+j} \end{array}$$

with *s* and α respectively as order and coefficients of the adopted BDF scheme, and *z* as a general variable with time derivative $\dot{z}$. Given the nature of the Kalman filter which estimates the states at time step k+1 from the estimated quantity at the previous time step *k*, a first order integrator has been adopted. From Equation (17), the first order integration of $\dot{x}$ takes the form:

(18) $$\begin{array}{c} {\dot{x}}_{k+1}=\alpha \left(x_{k+1}-x_{k}\right) \end{array}$$

The discrete-time scheme of Equation (2) becomes:

(19) $$\begin{array}{c} \left\{\begin{array}{c} {\dot{q}}_{k+1}-v_{k+1}+B{\left(q_{k+1}\right)}^{T}\mu_{s,k+1}=0\\ M\left(q_{k+1}\right){\dot{v}}_{k+1}+f\left(q_{k+1},v_{k+1}\right)-B{\left(q_{k+1}\right)}^{T}\lambda_{s,k+1}=0\\ {\dot{p}}_{k+1}=0\\ B\left(q_{k+1}\right)v_{k+1}=0\\ g(q_{k+1})=0 \end{array}\right. \end{array}$$

in which the first-order time derivatives are evaluated at the time-step k+1 by applying Equation (18). The zero^th^-order input dynamic is also included in the third row of Equation (18), where $\dot{p}\in R^{p}$ and *p* is the number of inputs. The final goal of this work is to estimate or predict jointly the state vectors x and $\dot{x}$, and the external loads identified in p, which will not be considered anymore as an input to the system, but another set of quantities to be estimated. This is the field of application of the augmented Kalman filter (AKF), which defines an augmented state vector $x_{a}$ and its time-derivative:

(20) $$\begin{array}{c} x_{a}={\left[q,v,p,\lambda_{s},\mu_{s}\right]}^{T} \end{array}$$

(21) $$\begin{array}{c} {\dot{x}}_{a}={\left[\dot{q},\dot{v},\dot{p},{\dot{\lambda }}_{s},{\dot{\mu }}_{s}\right]}^{T} \end{array}$$

The filter estimates both variables; thus, a new vector is introduced: ${\bar{x}}_{a}={\left[x_{a}^{T},{\dot{x}}_{a}^{T}\right]}^{T}$. An important feature of the AKF is that it considers the inaccuracies of model and observations, which are respectively identified in zero-mean Gaussian noise $w_{{\bar{x}}_{a}}$ and $w_{y}$. A covariance matrix associated with each noise vector is defined as $Q=E\left[w_{{\bar{x}}_{a}}w_{{\bar{x}}_{a}}^{T}\right]$, $R=E\left[w_{y}w_{y}^{T}\right]$.

**Figure 3:**
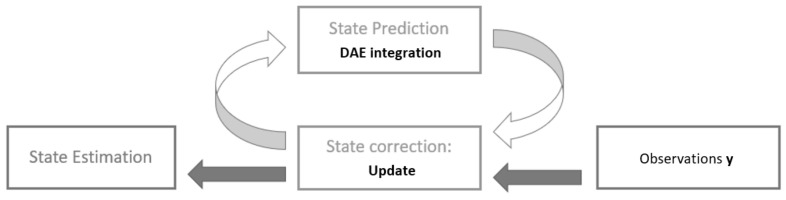
Kalman filter scheme based on the prediction-correction principle.

At this stage, all the ingredients needed to apply the AKF to the DAE system have been described. The estimation process can be divided into the two following steps, shown in Figure 3:

1. *Prediction*—integration of the equation of motion (19) by using the first-order BDF integrator scheme in Equation (17).
2. *Correction*—updating of the solution of the previous step with the available observations y:
  (22) $$\begin{array}{c} {\bar{x_{a}}}_{k+1}^{+}={\bar{x_{a}}}_{k+1}^{-}+K_{k+1}\left(y_{k+1}-k\left({\bar{x_{a}}}_{k+1}^{-}\right)\right) \end{array}$$
  where K is the Kalman gain, obtained in the definition of the filter by minimizing the estimation covariance. Further details on the derivation of K and on the update of the covariance matrices during the estimation can be found in [16].

## 3. Workflow for Estimation of States and Loads on a Rotor Blade

In the previous section, all the methodologies needed to approach the estimation problem for a coupled multibody-aerodynamic rotating system have been presented. As stated in the introduction, and indicated in Equation (5), the aerodynamic loads are functions of the states of the system, and in particular of the velocity and orientation vector of the airfoil section where the aerodynamic contribution is evaluated. The estimation problem could thus be approached in two ways:

1. Writing the vector p in Equation (19) as a function of q and $\dot{q}$, i.e., the orientation and velocity of a given point;
2. Modeling the aerodynamic forces as external loads without explicit dependence on the states.

Both options are feasible, depending on which problem we want to solve. In the first case, a simple KF can be applied, and only the state vector x is estimated. This option is feasible but can hardly reach high accuracy, particularly when a low-fidelity model is used. The second option is the case of the AKF; it is the most suitable option in the present cases, since it can compensate for modeling errors at the expense of the quality of the estimated loads. The question that arises is: how does this approach take into account the underlying aerodynamic model? In this case the aerodynamic model, coupled with the structural one, is needed only as a *reference case*. That means it allows the user to generate the data used as the input of the filter, i.e., the *observations*. Most importantly, the knowledge of the aerodynamic model, referring to the observations, is needed to post-process the estimation data and extract the correct aerodynamic loads.

**Figure 4:**
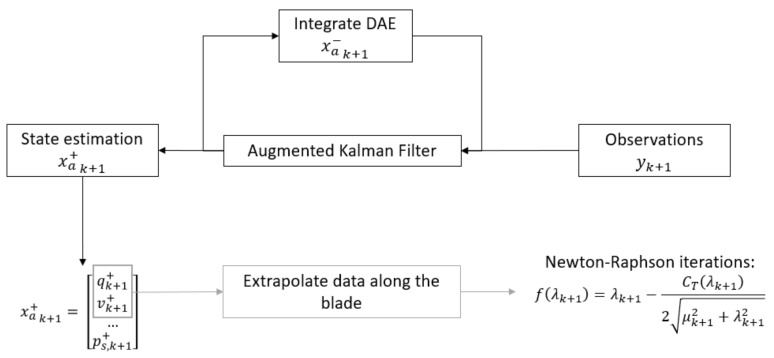
Proposed workflow for distributed aerodynamic loads, based on Kalman filtering estimation.

**Figure 5:**
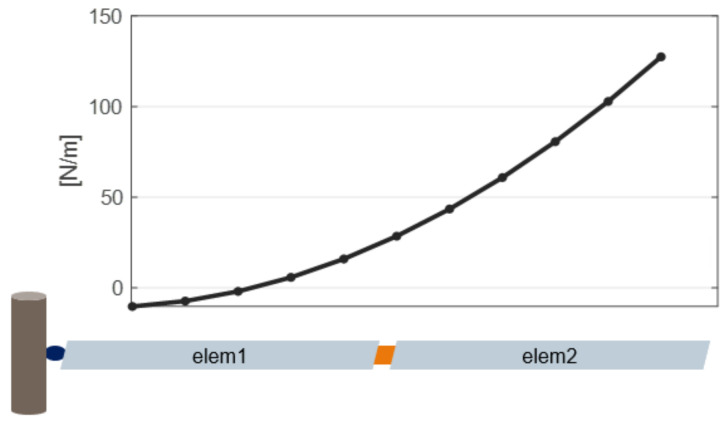
Example of a blade discretized with 2 elements. The lift distribution per unit length is shown above.

The proposed approach is schematically shown in Figure 4. It will be explained in the following through an example. Consider a blade discretized with two rigid bodies (Figure 5) and *(i)* modeled as indicated in the formulation of Section 2.1.1, or *(ii)* with aerodynamic loads acting at the application point of each body (usually at the quarter chord of the section). It is worth noticing that the distributed aerodynamic loads are evaluated on a more refined fictitious aerodynamic line (Figure 5) or surface, and the equivalent aerodynamic loads *L*, *D* and *M* (namely lift, drag, and the pitch moment) are applied on the rigid bodies. If a flexible finite element blade is considered, a more complex approach is needed to reduce the aerodynamic forces distribution on the structural model; this represents a possible future development of the present work. The preparation of the estimation process can be summarized as:

- *Collection of measurements that make the system observable*. This is an important requirement for Kalman-based techniques. For load estimation, it more strictly translates into a number of position-level observations equal to or greater than the number of loads to be estimated [41,43].
- *Definition of the set of loads.* This decision should be made in conjunction with the previous step. For multiple distributed loads, the definition of a subset of loads p could be useful to reduce the number of observations needed. For the case in Figure 5, the full input vector is $p=[L_{1},D_{1},M_{1},L_{2},D_{2},M_{2}]$; a good choice could be the selection of loads close to the tip $p_{s}=\left[L_{2},D_{2},M_{2}\right]$, because their contributions to the dynamics of the blade are higher.

The AKF will estimate the data $q_{1}^{+}$, $v_{1}^{+}$, $q_{2}^{+}$, $v_{2}^{+}$, together with the chosen subset of equivalent loads comprising the compensation of modeling errors $p_{s}^{+}=\left[L_{2}^{+},D_{2}^{+},M_{2}^{+}\right]$. The results of similar studies in other fields of application can be summarized as:

(23) $$\begin{array}{c} q^{+}=q+\epsilon_{q} \end{array}$$

(24) $$\begin{array}{c} v^{+}=v+\epsilon_{v} \end{array}$$

(25) $$\begin{array}{c} p^{+}=p+\epsilon_{p} \end{array}$$

with $\epsilon_{q}$ and $\epsilon_{v}$ at small and acceptable values, while $\epsilon_{p}$ assumes higher values as much as the modeling errors do. At this stage, the states at position and velocity level are accurate enough and can be re-used to evaluate the distributed lift contribution of Equation (5). Two steps are thus proposed:

- *Extrapolation of $\dot{q}$ along the blade span*. The components of the velocity vector along the blade can be approximated as a linear function of the position *r*:
  (26) $$\begin{array}{cc} \; & \dot{q}\left(r\right)i_{2}=\Omega r \end{array}$$
  (27) $$\begin{array}{cc} \; & \dot{q}\left(r\right)i_{3}=\dot{\beta }r \end{array}$$
- *Evaluation of the lift distribution along the blade span*. Recalling Equation (5), and given a certain flight condition with known pitch angle θ, the only unknown in the elemental lift will be the inflow angle ϕ:
  (28) $$\begin{array}{c} dL=dL(\lambda_{i}) \end{array}$$
  The iterative process in Section 2.2 can thus be applied to find the exact value of $\lambda_{i}$ at each time-step.

## 4. Numerical Validation on a 2-Blade Rotor Model

This section presents a numerical example of the methodology presented in Section 3 on a 2-blade rotor model. Python [44] multibody code with embedded aerodynamic force evaluation was used. Each blade was modeled as composed by four rigid bodies, connected through elastic elements with properties listed in Table 1 and uniform chord. No twist angle was considered. Two different experiments were done:

- Noisy reference observation data generated in Python code by simulating the coupled structural-aerodynamic model. Estimation of a subset of lumped loads on the same structural model without aerodynamic and subsequent distributed load evaluations.
- Reference observation data generated in MBDyn on a similar, but independently-formulated structural-aerodynamic model. Estimation of a subset of lumped loads on the structural Python model and subsequent evaluation of distributed loads.

One flight condition is shown in this contribution for the two cases: rotational speed Ω=60 rpm and pitch angle θ=3 deg. The airfoil used in the aerodynamic solver was a symmetric NACA0012 profile. To reduce the complexity of the entire problem, in this initial work the hub was fixed to the ground. Gravity was also applied.

### 4.1. Kalman-Based Estimation

The estimation process first requires the collection of a set of observations/measurements to be used as reference data. In a real test case, this means that a sensor layout needs to be designed satisfying the observability conditions, which allows for a robust estimation. As stated in Section 3, the first step is thus the definition of which states in terms of modes, loads or parameters the user wants to estimate. The scope of the AKF is the estimation of the external loads p, together with the states of the system. The normal modes of a structural dynamic model are all damped, resulting in an asymptotically stable system, a sufficient condition when the target is the estimation of the input loads. For further details on observability and stability requirements of the Kalman filter, refer to [43].

**Figure 6:**
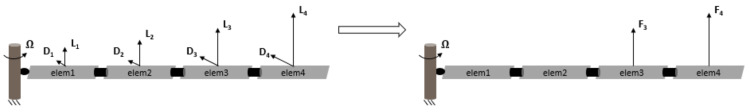
A 4-element blade model with all aerodynamic forces (**left**) and a reduced set of significant forces (**right**).

Considering a single blade as represented in Figure 6, a total of eight equivalent loads *L* and *D* are defined in the model. If the NACA airfoil is not symmetric, the pitch moment for each element has to be added. As explained in Section 2.3, a number of position-level sensors equal to or greater than the number of loads to be estimated is needed. For two blades modeled as in Figure 6, this means the usage of at least eight position sensors. If a more refined blade with a higher number of elements is modeled, the number of required sensors becomes unreasonable from a practical point of view, i.e., installation. As proposed in Section 3, a further simplification is adopted in this study by estimating a reduced set of loads acting on the most sensitive sections of the blade. Assuming that:

- The aerodynamic loads give higher contribution in the proximity of the blade tip;
- The in-plane loads are much smaller than the out-of-plane ones, namely, L≫D,

then the selection falls on two generic forces acting on elem3 and elem4, as shown in Figure 6. The subset of loads indicated as $p_{s}$ will be: $p_{s}={\left[F_{3},F_{4}\right]}^{T}$.

#### 4.1.1. Reference Noisy Data

Starting from the above-mentioned assumptions, the sensors’ layout in Table 2 is defined. *x*, *y* and *z* are the directions in the fixed reference frame. The selection was performed considering the forces modeled on *elem3* and *elem4* and thus the sensors were located on the same elements because they were considered to be more sensitive. Moreover, two velocity sensors were also included in order to capture the flapping motion. For a more complex system, an observability study is needed and it will be the focus of further development of this work.

Starting from this layout, a coupled multibody-aerodynamic simulation was performed and position/velocity sensor data were stored by applying additive white Gaussian noise, which defines the covariance matrix R. The missing information was on the model covariance Q. In previous work [25,27], a higher source of uncertainties was considered on the load states, such that $Q_{p}\gg Q_{x}$. This was a valuable assumption, since we can assume that the model used was accurate enough, but no information on the external loads is available. The zero^th^-order input model in the discrete time formulation will be read as:

(29) $$\begin{array}{cc} \; & p_{k+1}=p_{k}+w_{p} \end{array}$$

with $w_{p}$ Gaussian noise associated with vector p, an important tuning parameter of the filter [25,27].

**Figure 7:**
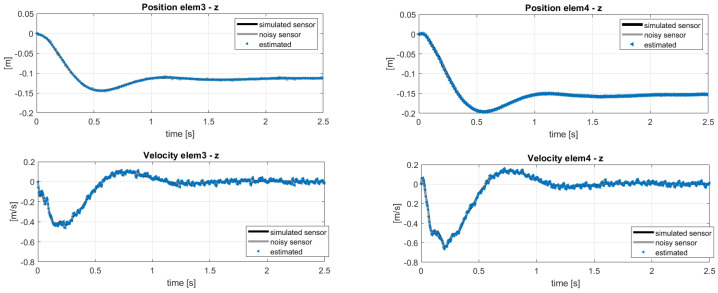
Estimation of the states used as sensors. Reference noisy data.

**Figure 8:**
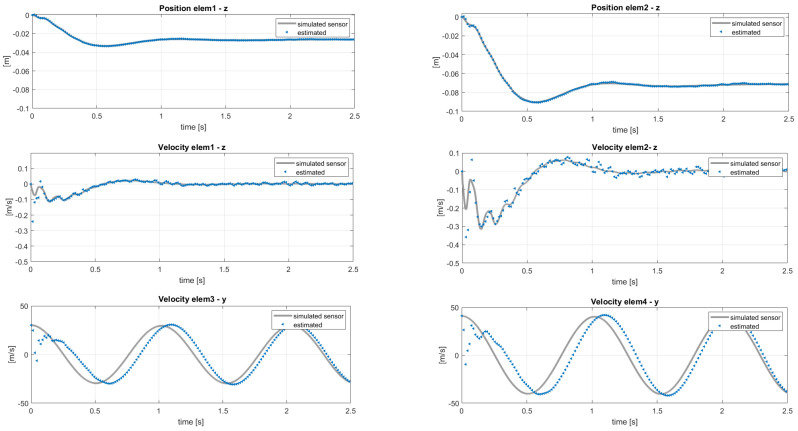
Estimation of the unmeasured velocity and position entities with reference to the sensor layout in Table 2. Reference noisy data.

The results of the estimation can be observed in Figure 7 and Figure 8 showing respectively the estimation of the states used as sensors and some of the unmeasured states along the blade. A good identification of the out-of-plane quantities is obtained, because the used sensors guarantee the observability of the flapping mode. Some delay in the time-histories of the in-plane estimation was instead observed, because of two assumptions made at the beginning:

- The sensor layout does not include in-plane quantities, e.g., position sensors in y-direction.
- Only out-of-plane loads are considered in the estimation, even if the reference sensors come from a coupled multibody-aerodynamic simulation which includes lift and drag forces. The choice to not estimate the drag forces was because the resulting magnitude would be comparatively small with respect to the computed lift.

**Figure 9:**
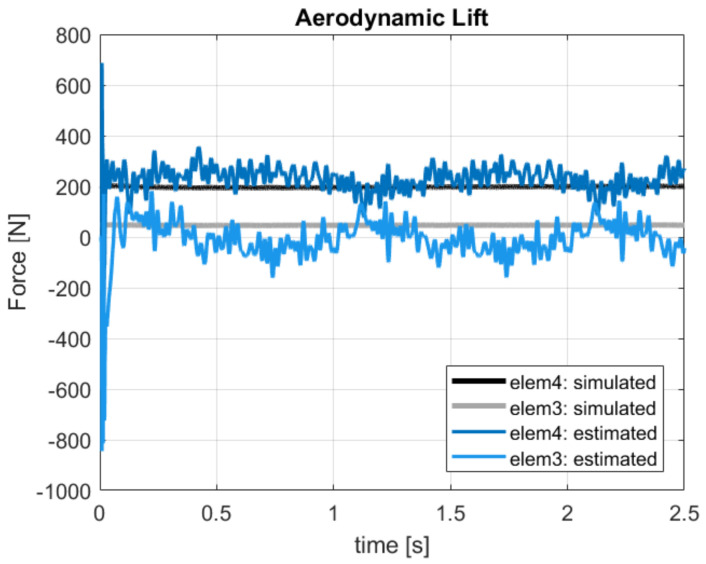
Force estimation on *elem3* and *elem4* with reference to the sensor layout in Table 2. Reference noisy data.

**Figure 10:**
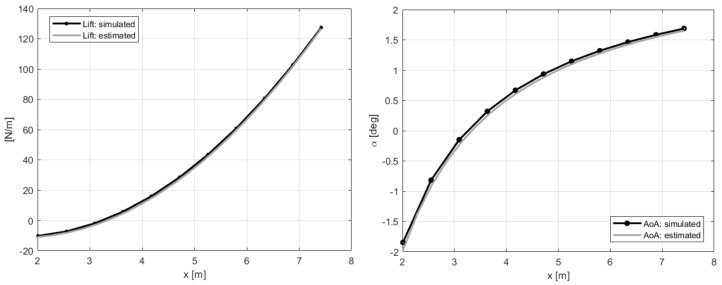
Distributed lift and angle of attack along the aerodynamic span in steady-state, with 11 points of integration. Reference noisy data used in the estimation.

**Figure 11:**
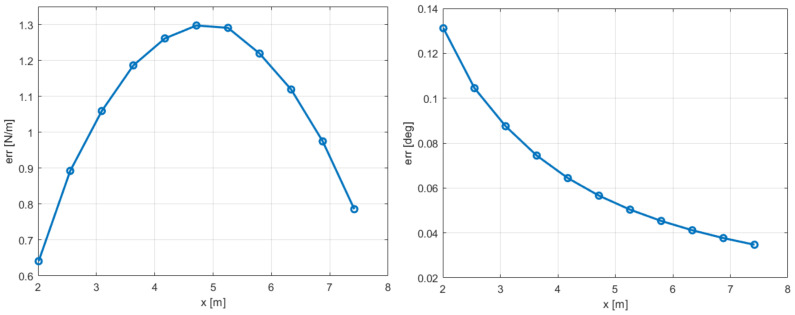
Absolute error of distributed lift and angle of attack—reference data in Figure 10.

These modeling errors were, however, compensated by the filter on the estimated loads shown in Figure 9. The real aerodynamic loads $L_{3}$ and $L_{4}$ evaluated in the simulation were compared with the estimated quantities $F_{3}$ and $F_{4}$, which showed mean values that did not match the reference ones. The good results to be considered are the ones on the states in Figure 8. At this point, the approach proposed in Section 3 and shown in Figure 4 could be executed. Performing an interpolation of the states ${\dot{q}}_{y}$ and ${\dot{q}}_{z}$ in order to extrapolate data on 11 points along the blade and applying the iterative Newton–Raphson method for inflow evaluation, the distributed lift field can be reconstructed. The results in terms of lift and angle of attack reached at the steady-state condition are shown in Figure 10. The absolute error, defined as the absolute value of the difference between simulated and estimated data, i.e., $err=|y_{s}-y_{e}|$, is shown in Figure 11.

#### 4.1.2. Reference Data from Mbdyn

In this section, the same analyses presented in Section 4.1.1 are performed. The difference is in the reference data used, which, in this case, were generated from MBDyn. The scope of this analysis is to further demonstrate the usability of the KF for a rotating blade, using data from an independent model as a different source of uncertainty. In the previous section, the uncertainty was introduced by adding noise on the reference data. In this case, the uncertainty is instead in the reference model itself, which presents a different formulation/modeling with respect to the one adopted in the Python solver. This framework is similar to a real test-case, where the numerical model usually cannot be perfectly correlated with the real one.

**Figure 12:**
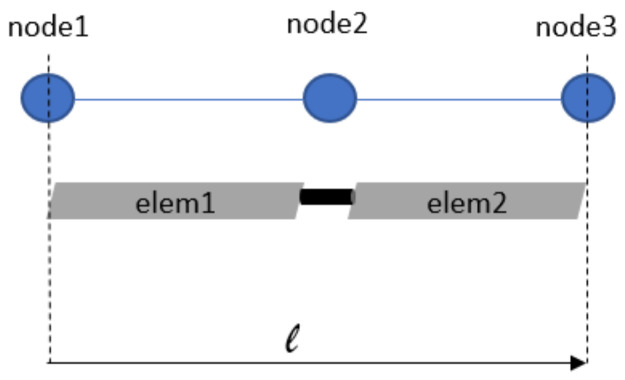
Graphic comparison between finite segment and finite volume modeling of a flexible element of free-length *l*.

The discrepancies encountered in the blade multibody modeling are in the finite segment formulation, which, for the MBDyn software, is replaced by the finite volume formulation [45]: the flexible beam element connects three nodes, i.e., bodies, and the internal forces are evaluated in well-chosen points between the two endpoints. Details can be found in the referenced paper. The different modeling also translates in a mismatch of mass distribution along the blade. A sketch of the two approaches for one flexible element of free-length *l* is shown in Figure 12. The relationship between rigid bodies and flexible elements is:

- Finite segment: $n_{bodies}=2\left(n_{flex}-1\right)$;
- Finite volume: $n_{bodies}=2n_{flex}+1$.

In the finite segment approach, two bodies are present in the length *l*; for the finite volume approach, three bodies are instead modeled. The total mass is the same for both formulations but a different distribution is observed.

**Figure 13:**
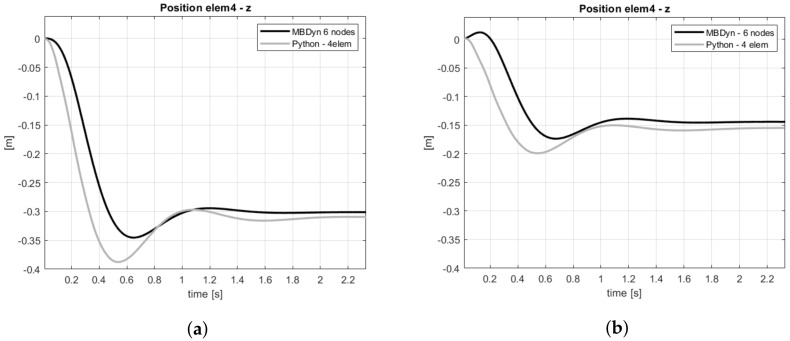
Comparison of vertical displacement at *elem4* position (**a**) without and (**b**) with aerodynamics. The model is in rotation and under gravity.

The reference blade in MBDyn has been modeled with the same properties listed in Table 1, seven rigid bodies and three flexible elements. This discretization was arbitrarily chosen, preserving the same number of flexible element used in the Python model. A comparison between the two models can be found in Figure 13a, which shows the vertical displacement evaluated at the same position of elem4 for both models, under gravity (applied gradually) and in rotating conditions. In Figure 13b, the same comparison is shown but also aerodynamic loads were used. The errors for the steady value are around 3% and 7%, and the transient regime shows a slightly different dynamic behavior.

**Figure 14:**
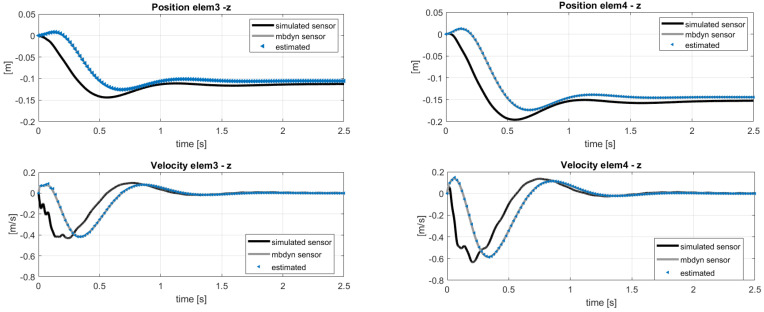
Estimation of the states used as sensors. Reference data from MBDyn.

**Figure 15:**
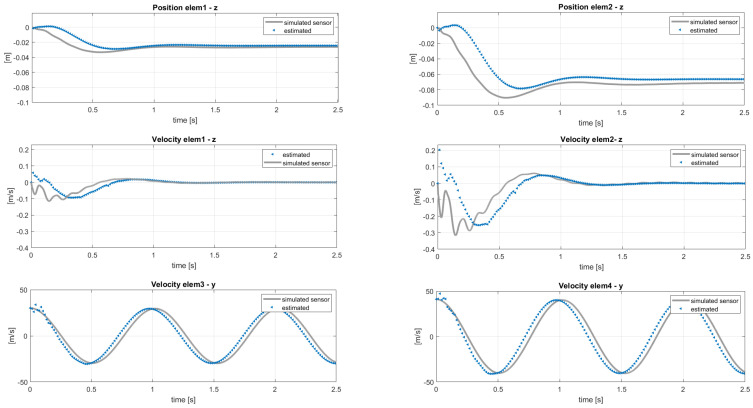
Estimation of the unmeasured velocity and position entities with reference to the sensor layout in Table 2. Reference data from MBDyn.

**Figure 16:**
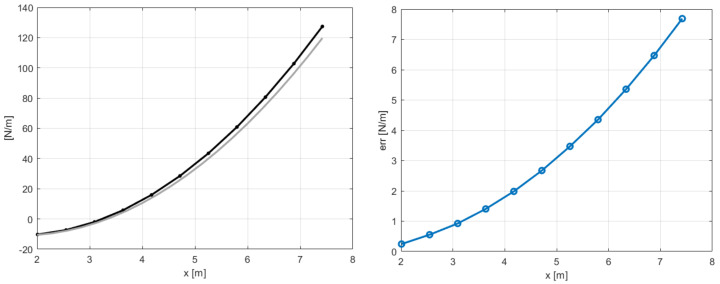
Distributed lift along the aerodynamic span in steady-state (**left**) and absolute error (**right**), with 11 points of integration. Reference data from MBDyn.

**Figure 17:**
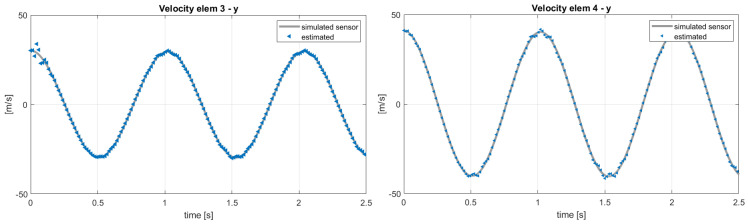
Estimation of the velocities of *elem3* and *elem4* with reference to the sensor layout in Table 3. Reference data from MBDyn.

Here, the scope of the application of the AKF is to compensate these modeling errors identified in a different response of the blade elements. The results of the same analysis performed in the previous section, with the sensor layout in Table 2, are shown in terms of estimated sensors and states in Figure 14 and Figure 15. In this case, the filter cannot compensate for both modeling and force errors and this is observed on the velocities in the *y* direction in Figure 15: these quantities should be the same in the two models, because the in-plane velocity is almost equal to Ωr for each position *r* along the blade span. By applying the post-processing iterative method for inflow evaluation, the distributed aerodynamic lift was obtained and shown in Figure 16 with the corresponding absolute error. The mismatch between the reference lift and the estimated one increases going from the root to the tip, because of its sensitivity to the in-plane velocity. In order to increase the accuracy of the estimation, a different sensor layout can be employed, including in-plane information of the blade, as listed in Table 3. With this set of sensors, the in-plane velocity can be correctly estimated and the results on elem3 and elem4 are shown in Figure 17.

## 5. Conclusions

In this paper, the problem of identifying states and aerodynamic loads on a rotating helicopter blade is addressed. A well-known and validated Kalman-based approach is proposed, as it has never been used before for this purpose in the rotorcraft field. A multibody articulated rotor with two blades is developed based on the finite segment theory, and a steady aerodynamic condition is considered with a uniform inflow model from the blade element momentum theory. One of the advantages of the Kalman filtering technique is in the usage of a reduced set of sensors which guarantee the observability of the system, or more specifically, of the estimated quantities. Although a thorough observability study has not been carried out because of the simplified model, the efficiency of the Kalman filter estimation has been proven by using simulated noisy sensors and reference data generated in MBDyn, independent multibody simulation software. Both cases have confirmed the conclusions made in previous work: when some modeling errors are present, e.g., structural properties or load location, all the uncertainties are compensated in the load estimation while still providing an accurate state reproduction. This is a great advantage of the Kalman filter in the augmented formulation over other techniques cited in the literature, reducing the need for highly accurate models, which are highly time-consuming to use, especially in the rotorcraft field. In the specific case of quasi-steady aerodynamic loads, the formulation of lift and drag is easily expressed in terms of the system states. Given a set of available sensor data, the proposed approach addressed the identification problem by modeling the aerodynamic loads as general external forces, while aiming to reach high accuracy only on states at position and velocity-level. This is already an advantage, because no aerodynamic evaluation is needed during the estimation. From the knowledge of the estimated states at each time step, an extrapolation of the velocity field data is proposed and the distributed lift will preserve only the dependence from the inflow velocity. At this stage, an implicit function can be iteratively solved. Velocity and position sensors are not feasible in a real test case, but the intent of the authors was to investigate the usage of other sensors, e.g., strain, and validate the presented work on experimental data. This research aims also to extend the proposed approach on a more complex system, by employing a flexible finite element blade model. In that case, the selection of the reduced set of loads will become a challenge in the estimation process.

## Figures and Tables

**Table 1:** Blade and rotor features.

Blade Structural Properties			Blade Elastic Properties		
Total mass	82.76	kg	$EA_{elem}$	5.69E8	N
Chord	0.537	m	$EI_{yy,elem}$	4.00E5	Nm${}^{2}$
Total Length	6.988	m	$EI_{zz,elem}$	4.00E5	Nm${}^{2}$
Radius	7.420	m	$GJ_{elem}$	8.40E5	Nm${}^{2}$/rad
**Root Hinges—Distance From Hub**			**Root Hinges—Elastic Properties**		
Flap offset	0.289	m	Flap damping characteristic	7.50E3	Nms/rad
Lag offset	0.269	m	Lag damping characteristic	7.00E3	Nms/rad
Pitch offset	0.432	m			

**Table 2:** Sensor layout.

Position Sensors		Velocity Sensors	
elem3	z	elem3	z
elem4	z	elem4	z

**Table 3:** Sensors layout.

Position Sensors		Velocity Sensors	
elem3	z	elem3	z
elem4	z	elem4	z
elem4	y		
